# Associations of Ficolins With Hematological Malignancies in Patients Receiving High-Dose Chemotherapy and Autologous Hematopoietic Stem Cell Transplantations

**DOI:** 10.3389/fimmu.2019.03097

**Published:** 2020-01-28

**Authors:** Anna S. Świerzko, Mateusz Michalski, Anna Sokołowska, Mateusz Nowicki, Agnieszka Szala-Poździej, Łukasz Eppa, Iwona Mitrus, Anna Szmigielska-Kapłon, Małgorzata Sobczyk-Kruszelnicka, Katarzyna Michalak, Aleksandra Gołos, Agnieszka Wierzbowska, Sebastian Giebel, Krzysztof Jamroziak, Marek L. Kowalski, Olga Brzezińska, Steffen Thiel, Misao Matsushita, Jens C. Jensenius, Gabriela Gajek, Maciej Cedzyński

**Affiliations:** ^1^Laboratory of Immunobiology of Infections, Institute of Medical Biology, Polish Academy of Sciences, Łódz, Poland; ^2^Department of Hematology, Comprehensive Cancer Center and Traumatology, Copernicus Memorial Hospital, Łódz, Poland; ^3^Department of Bone Marrow Transplantation and Oncohematology, Cancer Center and Institute of Oncology, Gliwice Branch, Gliwice, Poland; ^4^Department of Hematology, Medical University of Łódz, Łódz, Poland; ^5^Department of Hematology, Institute of Hematology and Transfusion Medicine, Warsaw, Poland; ^6^Department of Immunology and Allergy, Medical University of Łódz, Łódz, Poland; ^7^Department of Rheumatology, Medical University of Łódz, Łódz, Poland; ^8^Department of Biomedicine, Aarhus University, Aarhus, Denmark; ^9^Department of Applied Biochemistry, Tokai University, Hiratsuka, Japan

**Keywords:** complement, *FCN1*, *FCN2*, *FCN3*, ficolin, hematopoietic stem cell transplantation (HSCT), multiple myeloma, lymphoma

## Abstract

A prospective study of 312 patients [194 with multiple myeloma (MM) and 118 with lymphomas (LYMPH)] receiving high-dose chemotherapy and autologous hematopoietic stem cell transplantation (auto-HSCT) was conducted. Ficolins are innate immune defense factors, able to distinguish between “self” “abnormal self,” and “non-self” and contribute to the elimination of the last two by direct opsonization and/or initiation of complement activation *via* the lectin pathway. Concentrations of ficolin-1, ficolin-2, and ficolin-3 in serially taken serum samples were determined as were the polymorphisms of the corresponding (*FCN1, FCN2*, and *FCN3*) genes. Serum samples were collected before conditioning chemotherapy, before HSCT, and once weekly post-HSCT (four to five samples in total); some patients were also sampled at 1 and/or 3 months post-transplantation. The control group (C) consisted of 267 healthy unrelated individuals. Median ficolin-1 and ficolin-2 (but not ficolin-3) levels in MM patients' sera taken before chemotherapy were lower (and correspondingly frequencies of the lowest concentrations were higher) compared with controls. That appeared to be associated with the malignant disease itself rather than with post-HSCT complications (febrile neutropenia, infections accompanied, or not with bacteremia). Higher frequencies of the *FCN1* genotype G/A-C/C-G/G (corresponding to polymorphisms at positions −542, −144, and +6658, respectively) and *FCN2* gene heterozygosity for the −857 C>A polymorphism were found among patients diagnosed with MM compared with the C group. Furthermore, *FCN2* G/G homozygosity (−557 A>G) was found more frequently and heterozygosity G/T at +6424 less frequently among LYMPH patients than among the healthy subjects. Heterozygosity for +1637delC mutation of the *FCN3* gene was more common among patients diagnosed with lymphomas who experienced hospital infections. Although no evidence for an association of low ficolin-1 or ficolin-2 with infections during neutropenia following chemotherapy before HSCT was found, we observed a possible protective effect of ficolins during follow-up.

## Introduction

Ficolins are multimeric lectins marked by fibrinogen-like and collagen-like domains. The former is directly involved in recognition of pathogen/danger-associated molecular patterns, while the latter enables complexing with mannose-binding lectin-associated serine proteases (MASP). Those attributes make ficolins' multifunctional innate immune defense factors able to distinguish between “self,” “abnormal self,” and “non-self” and contribute to the elimination of the last two by direct opsonization and/or initiation of complement activation *via* the lectin pathway. Although expressed in various cell types (ficolin-1/M-ficolin in the bone marrow, monocytes, and neutrophils; ficolin-2/L-ficolin in hepatocytes; ficolin-3/H-ficolin in hepatocytes, alveolar type II pneumocytes and ciliated bronchial cells), all human ficolins circulate in the blood and participate in the systemic immune response. Ficolin-1, present in lung macrophages, and ficolin-3 are able to act locally as well, in the respiratory system (1–7).

The role of the complement system in cancer is complex. It is involved in the elimination of apoptotic/necrotic/cancer cells and some carcinogenic pathogens thus contributing to the prevention of tumorigenesis (8, 9). On the other hand, complement-associated chronic inflammation may favor transformation of host cells, and sublytic complement activation may disturb cell signaling, promote cell proliferation, and activate proto-oncogenes (10–18). Anaphylatoxins (C3a, C5a) were demonstrated to induce epithelial–mesenchymal transformation (EMT), activate matrix metalloproteinases, and suppress the function of immune cells in the tumor microenvironment (13–15, 17–23). C5a is thought to contribute to angiogenesis (24). Furthermore, complement affects the patient's response to chemotherapy and contributes to mobilization of hematopoietic cells from the bone marrow to the peripheral blood. The latter (involving mannose-binding lectin, MBL) is of particular importance in patients treated with hematopoietic stem cell transplantation (HSCT) (25). Regarding possible direct involvement of ficolins in anticancer immunity, ficolin-2 was found to suppress EMT and metastasis of hepatocellular carcinoma (26). Furthermore, ficolin-2 was shown to trigger an antitumor effect *via* promoting M1 polarization of macrophages, enhancement of secretion of cytokines and reactive nitrogen species as well as enhancement of proliferation and cytotoxicity of antigen-specific CD8^+^ T cells (27). Ficolin-3, in association with IgM, was found to kill cancer cells (28). Finally, we reported the interaction of recombinant ficolin-3 with ovarian cancer cells and proposed the existence of a plasma factor preventing its recognition of eukaryotic cells (29).

Hematological cancers like multiple myeloma, lymphomas, or leukemias derive from various cells of the immune system. Patients are at a high risk of morbidity and mortality from infections due to severe immunosuppression caused by both the disease and therapy as well as damage of anatomical barriers (mucosal injuries) and disruption of the gut microbiota. Bloodstream infections are the most common, but numerous subjects are affected by pneumonia or alimentary tract infections (30, 31). Multiple myeloma (MM) is an incurable, relatively common plasma cell cancer. However, the treatment strategy including chemotherapy and autologous hematopoietic stem cell transplantation (auto-HSCT) often allows prolongation of life and contributes to better quality of life (32–34). Lymphomas (LYMPH) constitute heterogenous lymphoid malignancies, most commonly arising from the B cells (>40 subtypes; >80% of cases) but sometimes also from T or NK cell lineages. Some of them are aggressive with fatal prognosis, while others are curable. Auto-HSCT preceded by high-dose chemotherapy is often employed as standard treatment in patients with both Hodgkin's and non-Hodgkin's lymphomas (35–37).

The aim of our study was to investigate the possible association of ficolins with hematological cancers (MM, LYMPH) and with susceptibility to hospital infections after chemotherapy followed by auto-HSCT. Serum concentrations of ficolins were determined serially before chemotherapy, before HSCT, and once weekly till hospital discharge (additionally, in minority of patients, ~45 and 100 days after HSCT). Selected single nucleotide polymorphisms of the *FCN1* (ficolin-1), *FCN2* (ficolin-2), and *FCN3* (ficolin-3) genes were investigated. Most of them are known to affect the corresponding protein level and/or its activity. The *FCN1* variant alleles at positions −542 (G>A) and −144 (C>A) were associated with higher serum ficolin-1 concentrations while minor alleles at positions +6658 (G>A), +7895 (T>C), and +7959 (A>G)—with lower serum ficolin-1 concentrations (38). In the case of the *FCN2* gene, variant alleles for −986 A>G, −557 A>G, −64 A>C, and +6424 G>T were related to lower ficolin-2, while those for −602 G>A, −4 A>G, and +6359 C>T had an opposite effect (39–41). Furthermore, the presence of a T variant at position +6359 resulted in higher affinity to ficolin-2 ligands. On the contrary, T allele corresponding to +6424 G>T SNP was associated with lower affinity (39). The +1637 C>delC mutation of the *FCN3* gene led to rare ficolin-3 deficiency in delC/delC homozygotes and low levels of this protein in the sera of the heterozygotes (42).

This study was complementary to our previous paper concerning the role of complement-activating collectins and associated serine protease-2 (MASP-2) in the same cohort of patients and controls (43).

## Materials and Methods

### Patients and Controls

Three hundred and twelve patients suffering from hematological malignancies and undergoing auto-HSCT were recruited. This group included 194 persons diagnosed with multiple myeloma (MM; 95 females and 99 males; mean age: 58.9 ± 8.9 years) and 118 with Hodgkin's or non-Hodgkin's lymphomas (LYMPH; 46 females and 72 males, mean age: 49.3 ± 13.1). Basic demographic data were published previously (43). All MM patients were newly diagnosed while for LYMPH patients, auto-HSCT was a second-line treatment. The standard chemotherapy for MM patients was melphalan, usually at a dosage of 200 mg/m^2^ (MEL-200), but in some cases lower MEL-140 or MEL-100 was used (43). Majority of the LYMPH patients were treated with carmustine (300 mg/m^2^), etoposide (800 mg/m^2^), cytarabine (1,600 mg/m^2^), melphalan (140 mg/m^2^) (BEAM) or (less frequently)—with BeAM (carmustine replaced with bendamustine). In some MM and LYMPH cases, chemotherapy was combined with radiotherapy (43). During hospital stay, crucial clinical parameters like white blood cell (WBC) count, absolute neutrophil count (ANC), platelet (PLT) count, inflammatory markers' [C-reactive protein (CRP), fibrinogen (FBG), and procalcitonin (PCT)] levels, and incidence of complications [infections (associated with bacteremia or not), febrile neutropenia (FN); duration of fever >38°C] were recorded and used for the analyses (43).

The control group included 267 individuals (unrelated volunteers with no history of cancer, autoimmune diseases, or recurrence of infections; 174 females and 93 males; mean age: 48 ± 13; age range: 18–84) (43). The study was approved by the local ethics committee, and written informed consent from patients and controls was obtained. This work conforms to the provisions of the Declaration of Helsinki. More detailed characteristics of the patients and controls were given in our previous paper (43).

### DNA and Serum Samples

DNA and serum samples described previously were used (43). Briefly, from the majority of patients, blood for serum isolation was taken before chemotherapy (as was blood for DNA isolation, sample 1), before HSCT (sample 2: usually 3–7 days later), and once weekly till hospital discharge (sample 3: usually 7 days after HSCT, mean 7.0 ± 0.8; sample 4: usually 14 days after HSCT, mean 13.6 ± 1.2; in some patients, sample 5: 21 days after HSCT, mean 20.3 ± 2). A minority of the patients were additionally sampled ~45 days (mean 45.3 ± 7.6; sample 45) and/or at ~100 days (mean 104.1 ± 19.3; sample 100) after HSCT; more details were previously published by Swierzko et al. (43).

### *FCN1* Genotyping

Polymorphisms at positions +6658 (G>A; A218T; exon 8, rs148649884) and +7895 (T>C; S268P; exon 9, rs150625869) were investigated using PCR–RFLP procedures, essentially as described by Swierzko et al. (44).

Another SNP located in exon 9 (+7959 A>G; N299S; rs138055828) and two located in the promoter region (−542 G>A, rs10120023 and −144 C>A, rs10117466) were tested with PCR–RFLP as well, using in-house procedures. Briefly, PCRs were run on a C1000 Thermal Cycler (Bio-Rad, Hercules, CA, USA) using appropriate spanning primers, designed with the use of PRIMER3 software, http://bioinfo.ut.ee/primer3/ (Table 1).

**Table 1:** Sequences of primers, lengths of PCR products/restriction fragments and corresponding restriction enzymes used for PCR–RFLP procedures for the investigation of selected *FCN1* gene single nucleotide polymorphisms.

**SNP**	**Primers (forward/reverse)**	**PCR product (bp)**	**Enzyme**	**Restriction fragments (bp)**
−542 G>A rs10120023	5′-CCCAGAAAATTCAGGGTTTG-3′ 5′-TAACTTTCAAATAATTTACTCCATC-3′	148	Taq1 65°C, 24 h	123 + 25
−144 C>A rs10117466	5′-TGAAGAGTCCCCCAGCTCT-3′ 5′-GGAAACATCCTTTGAGATGGC-3′	150	BsuRI (HaeIII) 37°C, 24 h	130 + 20
+7959 A>G rs138055828	5′-CACTAGCAGGTGCATGTGGA-3′ 5′-CGACTGTCATGCTTCAAACCTTA-3′	177	Tru1I 65°C, 24 h	156 + 21

The PCR conditions were as follows:

95°C for 3 min, 59°C for 30 s, then 35 cycles (72°C for 30 s, 95°C for 30 s, 59°C for 30 s) plus final elongation at 72°C for 3 min. PCR products were treated with restriction enzymes [all coming from Thermo Fisher Scientific (Waltham, MA, USA) in buffers recommended by the producer; conditions given in Table 1]. Products corresponding to alleles G (position −542), C (−144), and A (+7959) underwent digestion, while those corresponding to alleles A, A, and G, respectively, remained intact. The lengths of the restriction fragments are presented in Table 1. In the preliminary experiments, genotypes have been confirmed with the help of sequencing (not shown).

### *FCN2* Genotyping

Five SNPs of the *FCN2* gene, located within its promoter region: −986 A>G (rs3124252), −857 C>A, −602 G>A (rs3124253), −557 A>G (rs3811140), and −553 A>G were investigated by direct sequencing. Briefly, PCRs were run on a C1000 Thermal Cycler (Bio-Rad, Hercules, CA, USA) using appropriate spanning primers (designed with the use of PRIMER3 software, http://bioinfo.ut.ee/primer3/):

forward: 5′-ATTGAAGGAAAATCCGATG-3′,
reverse: 5′-GAAGCCACCAATCACGAAG-3′,

under the following conditions: 94°C for 3 min, then 34 cycles (94°C for 45 s, 61°C for 50 s, 72°C for 60 s), and finally 72°C for 7 min. The PCR products were purified with the help of EPPiC—Enzymatic Post-PCR Immediate Cleanup (A&A Biotechnology, Gdynia, Poland). Samples thus prepared were directly used as templates for sequencing, performed using the GeneAnalizer-3000 sequencer (Applied Biosystems, Foster City, CA, USA), reverse primer, BrightDyeTerminator Cycle Sequencing kit (NimaGen BV, Nijmegen, The Netherlands), and BDX64 Sequencing Enhancement Buffer (MCLab, San Francisco, CA, USA) according to the manufacturer's instructions. Sequence electropherograms were visually inspected to confirm base calling at the SNP sites using novoSNP (45).

Other polymorphisms of this gene: −64 A>C (rs78654553), −4 A>G (rs17514136) (both located within promoter), +6359 C>T (rs175491193), and +6424 G>T (rs7851696) (both exon 8) were analyzed using allele-specific PCR or PCR–RFLP procedures as described previously (46).

### *FCN3* Genotyping

The presence of +1637delC frameshift mutation of the *FCN3* gene was investigated using PCR–RFLP procedure as previously described by Michalski et al. (47).

### Determination of Serum Concentrations of Ficolins

Serum ficolin-1 and ficolin-3 concentrations were determined by TRIFMA [as described by Wittenborn et al. (7)] and ELISA [according to Michalski et al. (47)], respectively. Ficolin-2 levels were measured by TRIFMA. Briefly, microtiter plates (Optiplate-384HB, Perkin Elmer, Waltham, MA, USA) were coated with antificolin-2 (ABS 005-16, BioPorto Diagnostics, Copenhagen, Denmark, 1 μg/ml). Plates were blocked with 0.1% BSA and then incubated with sera to be tested, pre-diluted 1:10. Biotinylated mAb (clone GN4, 1:100, Hycult Biotech, Uden, The Netherlands) and Eu^3+^-labeled streptavidin (Perkin Elmer) were used for detection. After incubation with the enhancement solution (Perkin Elmer), fluorescence values were measured using Varioskan Flash reader (ThermoFisher Scientific, Waltham, MA, USA). Serum from a healthy volunteer (ficolin-2 concentration: 3,500 ng/ml) was used as a standard.

“Low” and “high” values were arbitrarily based on 10 and 95th percentiles, respectively, determined for the control group. Consequently, concentrations <620 ng/ml (ficolin-1), <1,670 ng/ml (ficolin-2), and <12.9 μg/ml (ficolin-3) were considered “low,” while concentrations >2,900 ng/ml, >6,350 ng/ml, and >34.9 μg/ml, respectively, were considered “high.”

### Statistical Analysis

The Statistica (version 13.3, TIBCO Software) and SigmaPlot (version 12.0, Systat Software) software packages were used for data management and statistical calculations. The medians of protein concentrations were compared using the Mann-Whitney *U*-test. It was chosen because values were not normally distributed (not shown). Changes during hospital stay and after discharge were analyzed by Friedman's ANOVA test. The frequencies of low or high levels, as well as genotypes/alleles were compared by two-sided Fischer's exact test (or χ^2^ when appropriate). Correlations were determined by Spearman's test. *P*-values < 0.05 were considered statistically significant.

## Results

### *FCN1, FCN2*, and *FCN3* Gene Polymorphisms

All patients and controls were T/T and A/A homozygotes for +7859 T>C and +7959 A>G *FCN1* gene polymorphisms, respectively. Data concerning *FCN1* SNPs are given in Table 2. Although no statistically significant differences between groups were found when results for each polymorphic site were analyzed separately, the genotype G/A-C/C-G/G (positions −542, −144, and +6658) was more frequent among patients diagnosed with MM than among controls [7.2 vs. 3.1%, *p* = 0.0497, OR = 2.42, 95% CI (0.99–5.89)].

**Table 2:** Frequency of genotypes associated with selected *FCN1* single nucleotide gene polymorphisms.

**Polymorphism**	**Genotype**	**Group**		
		**C**	**MM**	**LYMPH**
−542 G>A (rs10120023)	G/G	95 (37)	69 (35.6)	45 (38.1)
	G/A	125 (48.6)	105 (54.1)	52 (44.1)
	A/A	37 (14.4)	20 (10.3)	21 (17.8)
−144 C>A (rs10117466)	C/C	98 (38.1)	75 (38.7)	48 (40.7)
	C/A	123 (47.9)	97 (50)	54 (45.8)
	A/A	36 (14)	22 (11.3)	16 (13.6)
+6658 G>A (rs148649884)	G/G	256 (99.6)	193 (99.5)	118 (100)
	G/A	1 (0.4)	1 (0.5)	0
	A/A	0	0	0

*Percentages are shown in parentheses*.

For the *FCN2* gene (Table 3), heterozygosity for −857 C>A and G/G homozygosity (−557 A>G) were more common in the MM group compared with the C group. The latter relationship was even stronger in the LYMPH patients (OR = 13.6; *p* = 0.005). Furthermore, heterozygosity (+6424 G>T) occurred less frequently among LYMPH patients than among healthy subjects (or MM patients). No greater differences were noted for other tested *FCN2* SNPs (Table 3).

**Table 3:** Frequency of genotypes associated with selected *FCN2* single nucleotide gene polymorphisms.

**Polymorphism**	**Genotype**	**Group**		
		**C**	**MM**	**LYMPH**
−986 A>G (rs3124252)	A/A	91 (36.1)	59 (29.5)	40 (34.2)
	A/G	131 (52)	109 (56.5)	58 (49.6)
	G/G	30 (11.9)	27 (14)	19 (16.2)
−857 C>A	C/C	251 (99.6)	187 (96.9)	114 (97.4)
	C/A	1 (0.4)	6 (3.1)^a^	3 (2.6)
	A/A	0	0	0
−602 G>A (rs3124253)	G/G	160 (63.5)	110 (57)	70 (59.8)
	G/A	78 (31)	76 (39.4)	40 (34.2)
	A/A	14 (5.6)	7 (3.6)	7 (6)
−557 A>G (rs3811140)	A/A	185 (73.4)	139 (72)	86 (73.5)
	A/G	66 (26.2)	52 (26.9)	25 (21.4)
	G/G	1 (0.4)	2 (1)	6 (5.1)^b^
−553 A>G	A/A	245 (97.2)	190 (98.4)	116 (99.1)
	A/G	7 (2.8)	3 (1.6)	1 (0.9)
	G/G	0	0	0
−64 A>C (rs78654553)	A/A	203 (78.7)	152 (78.4)	100 (84.7)
	A/C	54 (20.9)	41 (21.1)	18 (15.3)
	C/C	1 (0.4)	1 (0.5)	0
−4 A>G (rs17514136)	A/A	105 (40.9)	84 (43.3)	47 (39.8)
	A/G	118 (45.9)	95 (49)	51 (43.2)
	G/G	34 (13.2)	15 (7.7)	20 (16.9)
+6359 C>T (rs175491193)	C/C	99 (38.8)	81 (41.8)	38 (32.2)
	C/T	115 (45.1)	89 (45.9)	63 (53.4)
	T/T	41 (16.1)	24 (12.4)	17 (14.4)
+6424 G>T (rs7851696)	G/G	199 (77.4)	153 (78.9)	104 (88.1)
	G/T	58 (22.6)	40 (20.6)	14 (11.9)^c^
	T/T	0	1 (0.5)	0

*Percentages are shown in parentheses*.

a *p = 0.046, OR = 8.05, 95% CI (0.96–67.46) (vs. C)*.

b *p = 0.005, OR = 13.6, 95% CI (1.61–114.03) (vs. C)*.

c *p = 0.016, OR = 0.46, 95% CI (0.25–0.87) (vs. C); p = 0.046, OR = 0.5, 95% CI (0.26–0.97) (vs. MM)*.

None of the patients or controls had a homozygous deletion at position +1637 of the *FCN3* gene, therefore no case of primary ficolin-3 deficiency was found. The frequencies of C/– genotype did not differ significantly between the main groups (Table 4). However, heterozygosity was relatively common [9.7%, OR = 3.8, 95% CI (1.3–11.36), *p* = 0.017 vs. C group] among patients diagnosed with lymphomas who had infections during their hospital stay.

**Table 4:** Frequency of genotypes associated with *FCN3* gene mutation, at position +1637.

**Genotype**	**Group**		
	**C**	**MM**	**LYMPH**
C/C	250 (97.3)	188 (96.9)	110 (93.2)
C/–	7 (2.7)	6 (3.1)	8 (6.8)

*Percentages are shown in parentheses*.

### Serum Concentrations of Ficolins Before Chemotherapy

**Figure 1:**
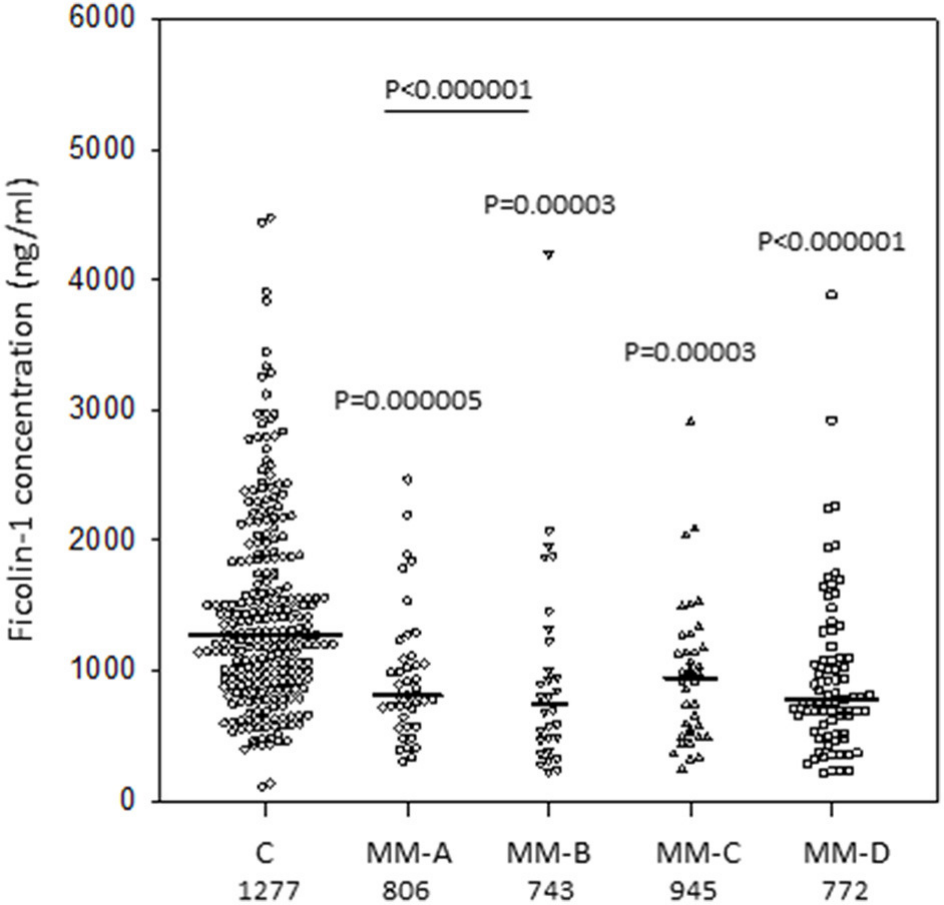
Serum concentrations of ficolin-1 in multiple myeloma patients (before conditioning chemotherapy) and controls. Bars present median values (given below group descriptions). MM-A, patients who experienced infections with proven bacteremia; MM-B, patients who experienced infections with no bacteremia; MM-C, patients who experienced febrile neutropenia; MM-D, patients who experienced none of afore-mentioned complications during hospital stay.

Median serum of ficolin-1 concentration in patients suffering from multiple myeloma (800 ng/ml; *n* = 187) was significantly lower than in healthy controls (1,277 ng/ml; *n* = 255; *p* < 0.000001), independently of complications recorded during hospital stay (Figure 1) or sex (not shown). The ROC analysis revealed relatively high differentiating potential of ficolin-1 between the MM patients and controls (curve area−0.72, *p* < 0.0001, cut-off−1,138 ng/ml, sensitivity−75.4%, specificity−62%). The median of ficolin-1 (as well as of ficolin-2 and−3) for patients diagnosed with lymphomas was not compared with the C or MM groups as their previous medication may affect their serum level. Consequently, low ficolin-1 levels (<620 ng/ml) were over-represented in the MM (31%) group in comparison with the healthy controls (10.2%) [*p* < 0.00001, OR = 4.14, 95% CI (2.47–6.93)]. No significant differences were found in the frequency of high (>2,900 ng/ml) concentrations (2.1 vs. 5.1%, respectively). Interestingly, we noted significantly higher median serum ficolin-1 level in LYMPH patients who experienced bacteremia (751 ng/ml) compared with those who had no complications during their hospital stay (371 ng/ml, *p* = 0.00009). As auto-HSCT was a second-line treatment for lymphoma patients and previous chemotherapy might affect concentrations of ficolins, data from them were not compared with the MM or C groups.

Although the median ficolin-1 was higher in female than in male controls (1,434 vs. 1,014 ng/ml; *p* < 0.000001), there was no significant difference within the MM group (806 vs. 764 ng/ml, respectively; *p* = 0.27).

**Figure 2:**
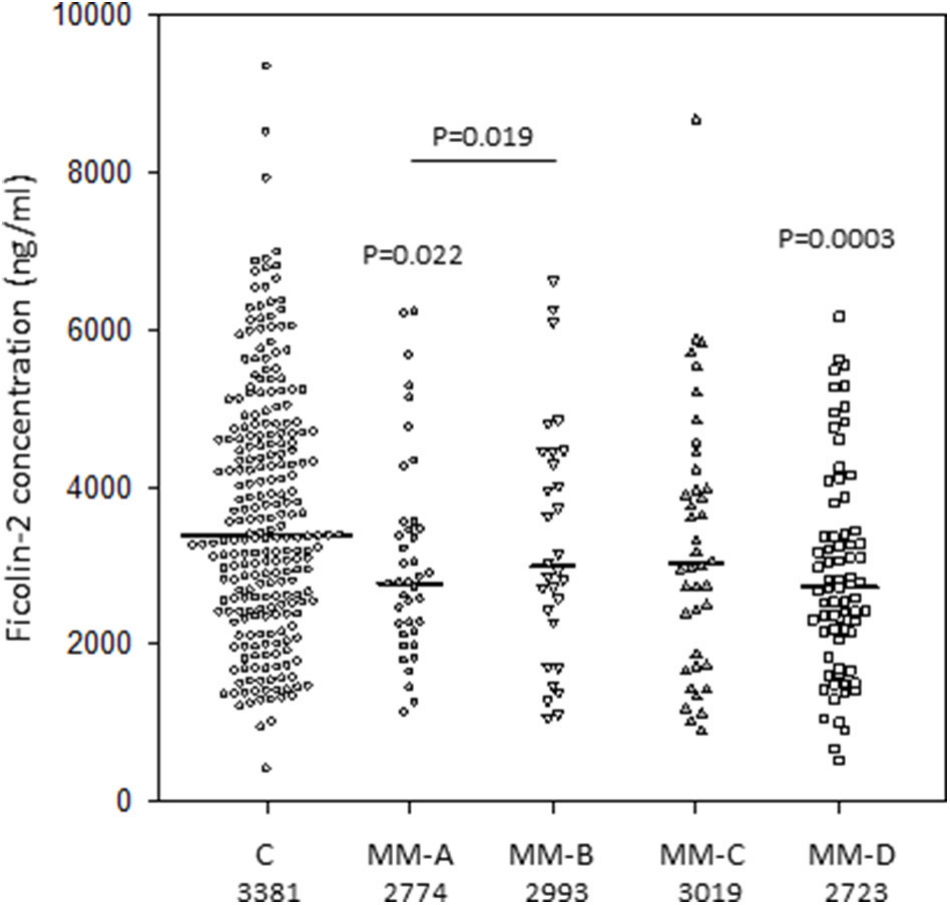
Serum concentrations of ficolin-2 in multiple myeloma patients (before conditioning chemotherapy) and controls. Bars present median values (given below group descriptions). MM-A, patients who experienced infections with proven bacteremia; MM-B, patients who experienced infections with no bacteremia; MM-C, patients who experienced febrile neutropenia; MM-D, patients who experienced none of afore-mentioned complications during hospital stay.

Similarly, ficolin-2 serum levels were lower in the MM patients (median: 2,825 ng/ml) than in controls (median: 3,381 ng/ml; *p* = 0.0001) (Figure 2). Ficolin-2 concentrations seemed not to predict particular complications after HSCT, and relationships were generally less significant than in the case of ficolin-1. Correspondingly, low levels (<1,670 ng/ml) were found more frequently among the MM subjects (17.5%) than among the healthy subjects [10%; *p* = 0.027, OR = 1.89, 95% CI (1.08–3.32)]. In contrast to ficolin-1, the median ficolin-2 was higher in men than in women among the healthy controls (4,269 vs. 3,076 ng/ml; *p* = 0.00001). However, for patients suffering from multiple myeloma (2,734 vs. 2,980 ng/ml; *p* = 0.11) there was no sex difference. ROC analysis of ficolin-2 revealed lower levels than ficolin-1 but still a significant differentiating potential between MM patients and healthy persons (curve area−0.61, *p* = 0.0001, cut-off−3,062 ng/ml, sensitivity−58.5%, specificity−60.9%).

**Figure 3:**
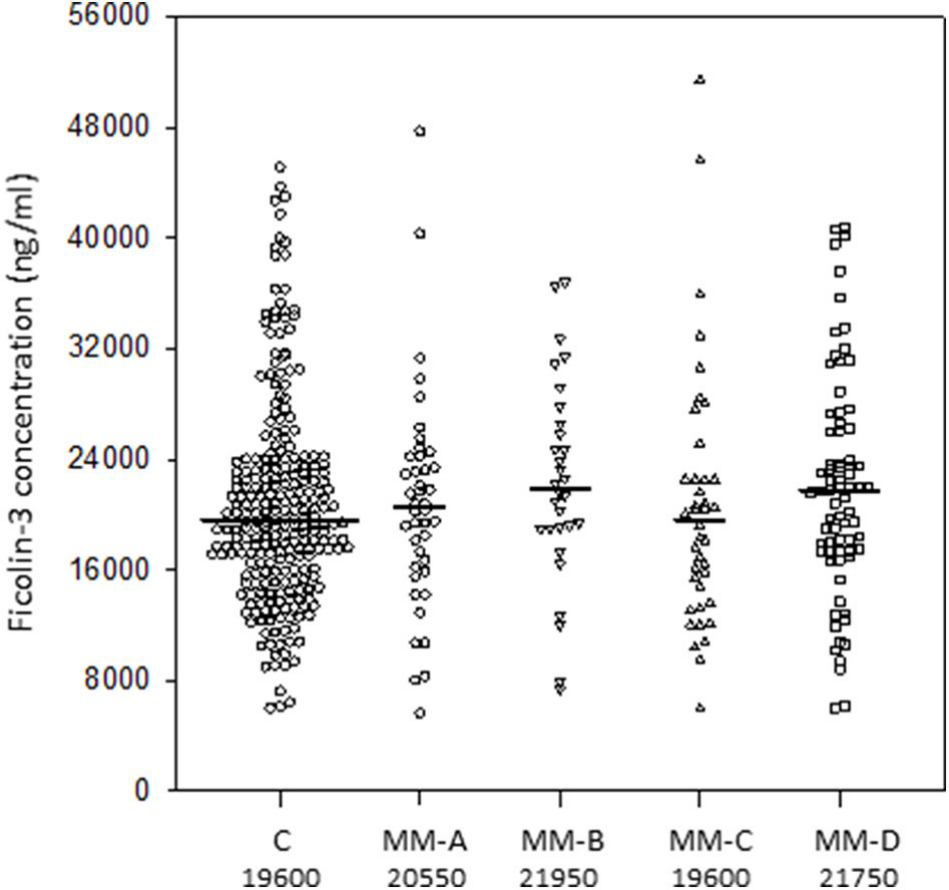
Serum concentrations of ficolin-3 in multiple myeloma patients (before conditioning chemotherapy) and controls. Bars present median values (given below group descriptions). MM-A, patients who experienced infections with proven bacteremia; MM-B, patients who experienced infections with no bacteremia; MM-C, patients who experienced febrile neutropenia; MM-D, patients who experienced none of afore-mentioned complications during hospital stay.

In contrast to the other members of the family, ficolin-3 levels did not differ significantly between the groups (Figure 3). As would be expected therefore, the MM patient group shared similar proportions of high and low values with the control group.

Median values of ficolin-3 were higher in males within the control (22.8 vs. 18.3 μg/ml, *p* < 0.000001), but this association was less marked in the MM (21 vs. 19.5 μg/ml, *p* = 0.061) patient group.

Interestingly, among the nine patients (of 161 followed-up for at least 6 months from the MM or LYMPH group) who experienced severe infections after hospital discharge, six (including three of four non-survivors) had low ficolin-1 serum concentration before chemotherapy, four (two non-survivors) had low ficolin-2, and four (two non-survivors) had low ficolin-3 levels. Two patients (one non-survivor and one survivor) were insufficient in all three factors, and four had low concentrations of two of the three ficolins (two non-survivors and two survivors).

### Changes in Serum Ficolins During Treatment

**Figure 4:**
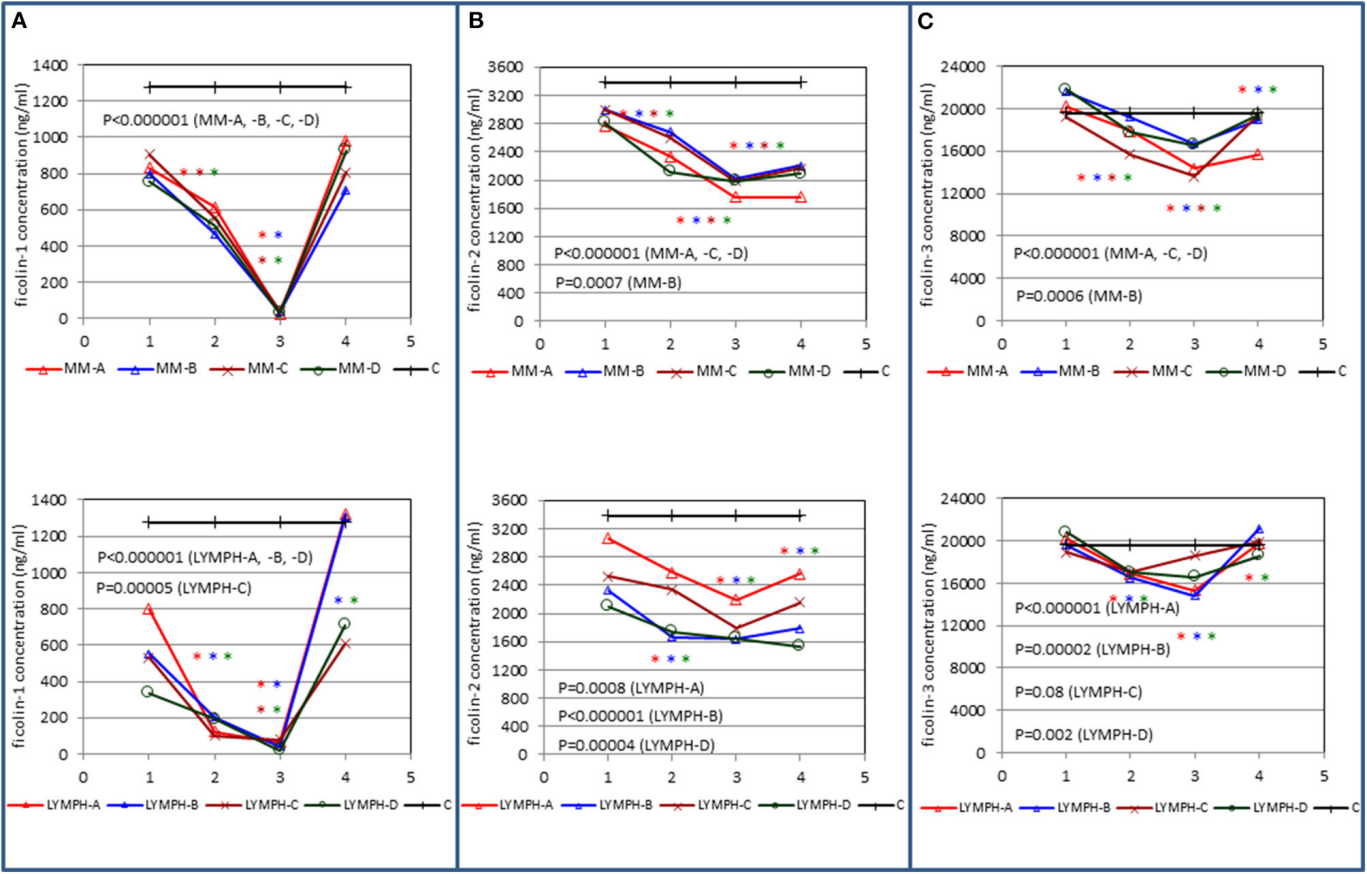
Changes in concentrations of ficolin-1 **(A)**, ficolin-2 **(B)**, and ficolin-3 **(C)** in patients, during treatment. 1—blood taken directly before conditioning chemotherapy; 2— blood taken directly before autologous hematopoietic stem cell transplantation; 3—blood taken 1 week after HSCT; 4—blood taken 2 weeks after HSCT. Data from sample 5 (3 weeks after HSCT) are not shown due to relatively low number of cases within each subgroup. Median values for each time-point are presented. MM-A, LYMPH-A, patients who experienced infections with proven bacteremia; MM-B, LYMPH-B, patients who experienced infections with no bacteremia; MM-C, LYMPH-C, patients who experienced febrile neutropenia; MM-D, LYMPH-D, patients who experienced none of afore-mentioned complications during hospital stay. C—controls (sampled once). Statistics: given *p*-values regard to Friedman's ANOVA while asterisks (in colors corresponding to curves) mark significant differences in comparison with sample 1 (Wilcoxon's paired sample test). Graphs show data from complete sets (levels of ficolins measured in all one to four samples) only.

Serum concentrations of all three ficolins underwent considerable changes in both the MM and LYMPH groups (Figure 4). In the MM group, a marked drop in median ficolin-1 level (by ~30–40%) was observed in the sample taken after chemotherapy (immediately before HSCT) and a week later reached a minimum (often undetectable in sample 3) which was correlated with WBC count. Within the next week, serum ficolin-1 was practically restored (no significant difference between medians for samples 4 and 1) (Figure 4A). It continued to increase with time (Figure 4A) returning to its initial value after ~3 months.

Corresponding data from the LYMPH group were broadly similar, but there was a more rapid drop (from ~42% in patients with no complications to almost 85% in patients who developed bacteremia) from the first to the second samples (Figure 4A). Again, ficolin-1 was often undetectable in sample 3, but a week later it had returned close (or was even higher) to its initial value. Like the MM group, 3 months post-HSCT, the average protein level was similar to that determined before chemotherapy.

Changes in ficolin-2 and ficolin-3 levels, although qualitatively similar, provided much flatter profiles (Figures 4B,C). Perhaps surprisingly, follow-up significantly lowered ficolin-2 and increased ficolin-3 one and three months post-HSCT in both MM and LYMPH groups (not shown).

The changes in ficolin-1 illustrated in Figure 4A correlated strongly with leukocyte numbers and moderately with platelet count in both groups of patients (Table 5). Ficolin-3 exhibited similar but weaker relationships which were nonetheless statistically significant. Changes in ficolin-2 also correlated weakly but significantly with leukocytes and platelets for the MM patients but only with platelets in the LYMPH group. These and certain inverse relationships with inflammatory markers are detailed in Table 5.

**Table 5:** Correlations (Spearman's) between variations (samples 1–5) of concentrations of tested proteins and selected clinical parameters (significant positive and inverse correlations marked with blue and red, respectively).

**Tested factor**	**Blood cell counts**			**Markers of inflammation**			**MASP-2**
	**WBC**	**ANC**	**PLT**	**CRP**	**FBG**	**PCT**	
**(A)**							
Ficolin-1	*r* = 0.58	*r* = 0.52	*r* = 0.41	*r* = −0.09	*r* = −0.52	*r* = 0.49	*r* = −0.08
	*p* < 0.000001	*p* < 0.000001	*p* < 0.000001	*p* = 0.67	*p* = 0.0001	*p* = 0.03	*p* = 0.02
	*n* = 791	*n* = 783	*n* = 789	*n* = 517	*n* = 49	*n* = 20	*n* = 842
Ficolin-2	*r* = 0.16	*r* = 0.14	*r* = 0.28	*r* = −0.2	*r* = −0.26	*r* = −0.56	*r* = −0.09
	*p* = 0.00001	*p* = 0.00007	*p* < 0.000001	*p* = 0.000004	*p* = 0.069	*p* = 0.01	*p* = 0.012
	*n* = 783	*n* = 775	*n* = 781	*n* = 511	*n* = 49	*n* = 20	*n* = 835
Ficolin-3	*r* = 0.17	*r* = 0.13	*r* = 0.21	*r* = −0.14	*r* = −0.28	*r* = 0.04	*r* = 0.35
	*p* = 0.000001	*p* = 0.0004	*p* < 0.000001	*p* = 0.002	*p* = 0.055	*p* = 0.87	*p* < 0.000001
	*n* = 791	*n* = 783	*n* = 789	*n* = 517	*n* = 49	*n* = 20	*n* = 843
**(B)**							
Ficolin-1	*r* = 0.69	*r* = 0.56	*r* = 0.36	*r* = −0.22	*r* = −0.26	*r* = 0.19	*r* = −0.18
	*p* < 0.000001	*p* < 0.000001	*p* < 0.000001	*p* = 0.0001	*p* = 0.027	*p* = 0.2	*p* = 0.00003
	*n* = 463	*n* = 443	*n* = 462	*n* = 302	*n* = 72	*n* = 48	*n* = 542
Ficolin-2	*r* = 0.07	*r* = −0.02	*r* = 0.18	*r* = −0.08	*r* = −0.18	*r* = −0.06	*r* = 0.03
	*p* = 0.11	*p* = 0.72	*p* = 0.00008	*p* = 0.14	*p* = 0.14	*p* = 0.68	*p* = 0.45
	*n* = 462	*n* = 442	*n* = 462	*n* = 301	*n* = 72	*n* = 48	*n* = 541
Ficolin-3	*r* = 0.18	*r* = 0.14	*r* = 0.14	*r* = −0.13	*r* = −0.3	*r* = 0.49	*r* = 0.18
	*p* = 0.00009	*p* = 0.003	*p* = 0.003	*p* = 0.02	*p* = 0.01	*p* = 0.0004	*p* = 0.00002
	*n* = 463	*n* = 443	*n* = 462	*n* = 302	*n* = 72	*n* = 48	*n* = 542

***(A)** patients suffering from multiple myeloma; **(B)** patients suffering from lymphomas*.

*WBC, white blood cell count; ANC, absolute neutrophil count; PLT, platelet count; CRP, C-reactive protein; FBG, fibrinogen; PCT, procalcitonin*.

Weak but highly significant correlations among the ficolins themselves were noted in both groups of patients (data not shown). Additionally, the levels of ficolin-1 correlated inversely, while ficolin-3 correlated positively with MASP-2 [reported previously by Swierzko et al. (43)]. In the case of ficolin-2, the inverse correlation with MASP-2 was found for the MM group only (Table 5).

## Discussion

The clinical associations of ficolins in the context of hematological malignancies and/or chemotherapy- and bone marrow transplantation-related infections have not been studied extensively. Schlapbach et al. (48) found significantly lower median ficolin-1 serum concentrations in children diagnosed with acute myeloid leukemia (AML) and acute lymphoblastic leukemia (but not lymphoma) than in controls. They also reported positive correlations of ficolin-1 level with peripheral blood leukocyte counts and proportions of both erythroid and myeloid precursors in the bone marrow but inverse correlations with leukemic blasts in the blood and bone marrow. Our results demonstrate significantly lower median ficolin-1 in MM patients compared with healthy controls, independently of complications occurring during hospitalization. We confirmed significant correlations of ficolin-1 with WBC counts (in LYMPH group as well) but extended the data by serial measurements during hospital treatment and follow-up. Changes in ficolin-1 reflected leukocyte counts, with the lowest (often undetectable) level 1 week after HSCT and full reconstitution 100 days post-HSCT. For the first time, we found a possible association of *FCN1* polymorphism with multiple myeloma: the genotype G/A-C/C-G/G (at positions −542, −144, and +6658, respectively) was more common among patients than among controls, although the difference only reached borderline significance. Schlapbach et al. (48) found no association of low ficolin-1 (defined as concentration <0.5 μg/ml) with febrile neutropenia accompanied or not with bacteremia in children undergoing anticancer chemotherapy. Interestingly, significantly higher median serum ficolin-1 level in LYMPH patients who experienced bacteremia compared with those who had no complications during their hospital stay was found. Such a difference was not observed in the MM group. In contrast, Ameye et al. (49) observed lower ficolin-1 concentrations in adult hematological cancer (leukemias, lymphomas, and others) patients undergoing chemotherapy who suffered from severe infections in comparison with patients who did not develop such infections. As we recently reported an association of high mannose-binding lectin levels with hospital-acquired infections in the multiple myeloma group (43), it seems that both MBL and ficolins have not only no protective role from pathogens within short period after chemotherapy but also they may contribute to some adverse effects.

Ficolin-2 serum levels were also lower in MM patients compared with controls and rather were not associated with particular post-chemotherapy/HSCT complications. However, ROC analysis revealed a relatively high (although lower than ficolin-1) potential of ficolin-2 concentration measurement to differentiate the patients from the healthy subjects. Concentration changes during hospitalization were not as striking as for ficolin-1 but generally correlated with the latter and with leukocyte counts. However, the shape of the curves differed from those reported earlier for complement activating collectins (MBL, CL-LK, determined in the same cohort) of hepatic origin (like ficolin-2) (43). Our data suggest moreover a possible association of some *FCN2* polymorphisms with multiple myeloma (−857 C>A) or lymphomas (−557 A>G; +6424 G>T). The results presented here seem to be in accordance with previous reports by Ameye et al. (49) and Kilpatrick et al. (50) who found no influence of its concentration on the risk of chemotherapy-related infections in adults. Later, Pana et al. (51) reported associations of GGACT, GGATG, AGACG, GGACG *FCN2* haplotypes (corresponding to SNPs at positions: −986 A>G, −602G>A, −4 A>G, +6359 C>T, and +6424 G>T, respectively, that have inconsistent influences on protein concentration) with prolonged FN episodes and bacterial infections in children diagnosed with B-cell ALL after chemotherapy. Ficolin-2 has been also considered as a biomarker for post-allo-HSCT sinusoidal obstruction syndrome (SOS) (52, 53).

Our data concerning ficolin-3 revealed no association with cancer. However, heterozygosity for the +1637delC mutation seemed to be associated with elevated risk for infections in the LYMPH group. The profile of changes of ficolin-3 levels in patients was generally similar to that demonstrated for ficolin-2. No impact of ficolin-3 on incidence of infections or febrile neutropenia in adult patients with hematological malignancies was previously reported by Ameye et al. (49), Kilpatrick et al. (50), and Islak Mutcali et al. (54). Low ficolin-3 concentration was however suggested to be a risk factor for febrile neutropenia (especially with bacteremia) in pediatric cancer patients treated with chemotherapy (55).

Generally, the differences in the serum levels of ficolins between patients and controls (significantly lower medians in the MM group) might be indicative of their direct clinical association and protective role. Such a hypothesis has been supported, at least to some extent, by data from genotyping. On the other hand, those differences seem rather to be the effect of protein consumption and/or dysregulated expression of the genes during carcinogenesis. The latter in turn may be supported by virtually the same ficolin-1 and−2 levels in male and female patients regardless of significantly higher ficolin-1 and lower ficolin-2 in women compared with men within the control group. Such differences observed in healthy individuals are in agreement with a previous report (56). Irrespective of the causal connection between low ficolin-1/ficolin-2 and disease, both factors might be considered as supplementary markers; that, however, needs to be confirmed in an independent study. It has to be taken into consideration that the type of blood collection tubes or sample handling procedures may affect ficolin-1 and ficolin-2 concentrations (57–59). Therefore, it is crucial to use the same procedures for all patients/controls. Highly significant correlations of ficolin-1 (synthesized in the bone marrow, monocytes, and neutrophiles) with WBC, ANC, and PLT were obvious, but similar relations were found also for factors of hepatic origin: ficolin-2 (although in the MM group only) and ficolin-3. Those findings supposedly reflect a hepatotoxic effect of chemotherapy and following liver regeneration, apparently correlated with bone marrow/leukocyte recovery. Furthermore, some inverse correlations with inflammatory markers (as CRP or FBG) were noted (Table 5). As concentrations of those markers were determined mainly in patients who experienced infections, it may result, at least partially, from consumption of ficolins during the acute phase response. Interestingly, reverse associations in the MM patients (inverse correlations with leukocyte counts and positive with CRP) were previously reported for complement-activating collectins (43) which suggest both their distinct from ficolins impact of chemotherapy on expression of genes and distinct response to infection.

This paper complements our recent report concerning collectins and MASP in the same cohort (43). Both papers documented possible associations of factors specific for the lectin pathway of complement (especially on genetic background) with multiple myeloma or lymphoma (MBL, ficolin-1, ficolin-2) or hospital infections (MASP-2, ficolin-3). We previously suggested that MBL deficiency is not associated with infections during the short period of neutropenia following conditioning treatment before HSCT, but it does during follow-up when active lectin is able to act in combination with recovered phagocytes (43). Data reported here (although from few patients only, experiencing the most severe infections) are consistent with a similar role for ficolins.

## Data Availability Statement

The raw data supporting the conclusions of this article will be made available by the authors, without undue reservation, to any qualified researcher.

## Ethics Statement

The study was approved by the Ethics Committee of the Medical University of Lodz. The written informed consent from patients/controls was obtained. This work conforms to the provisions of the Declaration of Helsinki.

## Author Contributions

MC, KJ, AW, and ASŚ co-authored the project and designed the study. ASŚ and MC planned and supervised the experimental work which was done by ASŚ, AS, MMi, AS-P, ŁE, and GG. AW, SG, and KJ supervised qualification and recruitment of patients and/or controls. MN, IM, AS-K, MS-K, KM, OB, and AG were responsible for patients' qualification, taking and collecting samples as well as collecting clinical data/follow-up. MK and AG qualified controls, provided DNA/serum samples and corresponding clinical data. ST and JJ produced antificolin-1 monoclonal antibodies and discussed the data. MMa produced antificolin-2 monoclonal antibodies and discussed the data. MC performed the statistical analysis. ASŚ revised the data. MC wrote the original draft of the paper. All authors contributed to the manuscript revision/correction and approved the version to be submitted.

### Conflict of Interest

The authors declare that the research was conducted in the absence of any commercial or financial relationships that could be construed as a potential conflict of interest.

## Abbreviations

HSCT: hematopoietic stem cell transplantation
LYMPH: lymphoma
MM: multiple myeloma.
